# Role of 14-3-3ζ in Platelet Glycoprotein Ibα-von Willebrand Factor Interaction-Induced Signaling

**DOI:** 10.3390/ijms13055364

**Published:** 2012-05-02

**Authors:** Weilin Zhang, Lili Zhao, Jun Liu, Juan Du, Rong Yan, Kesheng Dai

**Affiliations:** 1 School of Biological Science and Medical Engineering, Beijing University of Aeronautics and Astronautics, 37 Xueyuan Road, Haidian district, Beijing 100083, China; E-Mails: zhangwl@be.buaa.edu.cn (W.Z.); liiiiily@163.com (L.Z.); liujuno0o@sina.com (J.L.); djsmile1987@yahoo.com.cn (J.D.); 2 Jiangsu Institute of Hematology, The First Affiliated Hospital of Soochow University, Key Laboratory of Thrombosis and Hemostasis of Ministry of Health, Suzhou 215007, China; E-Mail: yanrongbaobao@163.com

**Keywords:** 14-3-3ζ, glycoprotein (GP) Ib-IX, platelets, von Willebrand factor (VWF)

## Abstract

The interaction of platelet glycoprotein (GP) Ib-IX with von Willebrand factor (VWF) exposed at the injured vessel wall or atherosclerotic plaque rupture initiates platelet transient adhesion to the injured vessel wall, which triggers intracellular signaling cascades leading to platelet activation and thrombus formation. 14-3-3ζ has been verified to regulate the VWF binding function of GPIb-IX by interacting with the cytoplasmic domains of GPIb-IX. However, the data regarding the role of 14-3-3ζ in GPIb-IX-VWF interaction-induced signaling still remain controversial. In the present study, the data indicate that the S609A mutation replacing Ser^609^ of GPIbα with alanine (S609A) significantly prevented the association of 14-3-3ζ with GPIbα before and after the VWF binding to GPIbα. GPIb-IX-VWF interaction-induced activations of Src family kinases and protein kinase C were clearly reduced in S609A mutation. Furthermore, S609A mutation significantly inhibited GPIb-IX-VWF interaction-induced elevation of cytoplasmic Ca^2+^ levels in flow cytometry analysis. Taken together, these data indicate that the association of 14-3-3ζ with the cytoplasmic domain of GPIbα plays an important role in GPIb-IX-VWF interaction-induced signaling.

## 1. Introduction

The interaction of platelet glycoprotein (GP) Ib-IX with von Willebrand factor (VWF) exposed at the injured vessel wall initiates platelet transient adhesion [1–3], and simultaneously triggers intracellular signaling cascades [4], such as activation of multiple protein kinases, elevation of intracellular Ca^2+^ levels, and phosphatidylserine (PS) exposure, leading to integrin α_IIb_β_3_ activation and integrin-dependent platelet stable adhesion and thrombus formation [5]. Although several typical events have been confirmed to play key roles in GPIbα-VWF interaction-induced platelet signaling, the molecule that initiates the GPIbα-VWF interaction-induced signaling leading to platelet activation remains unknown.

Several intracellular molecules that have been confirmed to interact with the cytoplasmic domain of the GPIb-IX complex are involved in platelet activation. Filamin A interacted with the cytoplasmic 557–579 sequence of GPIbα regulates tyrosine kinase signaling in platelets under high shear stress conditions [6–9]. Calmodulin binds directly to the juxtamembrane cytoplasmic sequences of GPIbβ and GPV in resting platelets, but it dissociates from GPIb-IX when platelets are activated [10]. Phosphoinositide 3-kinase (PI3-kinase) interacts with the cytoplasmic domain of GPIbα and is associated with GPIb-IX-mediated platelet functions [11]. The interaction of 14-3-3ζ with the cytoplasmic domains of GPIb-IX plays a key role in the VWF binding function of GPIb-IX and subsequent platelet activation [12–14]. However, the data regarding the role of 14-3-3ζ in GPIb-IX-VWF interaction-induced signaling still remain controversial [15–17]. It has been reported that deletion of the 14-3-3ζ binding site in the C-terminal cytoplasmic domain of GPIbα inhibited GPIb-IX-mediated α_IIb_β_3_ activation and cell spreading on VWF surface [15]. On the other hand, the data from another group indicated that binding of 14-3-3ζ to GPIbα inhibited platelet spreading on VWF surface, while disruption of 14-3-3ζ interaction with GPIbα increased integrin-induced cytoskeletal reorganization [16]. Therefore, the role of 14-3-3ζ in GPIb-IX-VWF interaction-induced platelet activation needs to be further investigated.

In the current study, the data show that disruption of 14-3-3ζ interaction with GPIbα by the S609A mutation induced inhibition of GPIb-IX-VWF interaction-induced signaling cascades.

## 2. Results

### 2.1. The S609A Mutation Disrupts the Association of 14-3-3ζ with GPIbα before and after VWF Binding to GPIbα

**Figure 1:**
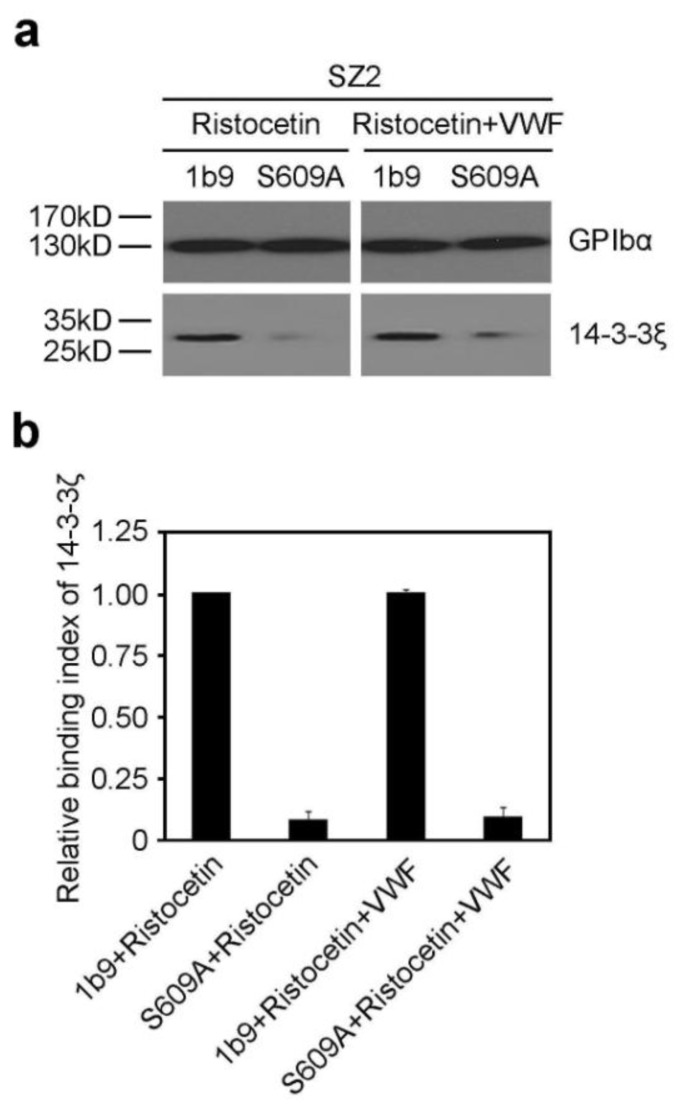
The S609A mutation disrupts the interaction of 14-3-3ζ with GPIbα before and after von Willebrand factor (VWF) binding. (**a**) 1b9 or S609A cells were stimulated by ristocetin in the presence or absence of VWF and solubilized in lysis buffer. The lysates were incubated with SZ2, and then precipitated with protein G beads. The precipitates were subjected to Western blot with SZ2 and anti-14-3-3ζ antibody, respectively. The immunoblot is representative of 3 independent experiments; (**b**) Quantitative data from 3 independent experiments (mean ± SD) are shown. The relative binding index of 14-3-3ζ equals arbitrary quantitation of 14-3-3ζ/GPIbα of treated cells divided by 14-3-3ζ/GPIbα of 1b9 cells stimulated by ristocetin in the absence of VWF.

14-3-3ζ has been confirmed to regulate the VWF binding function of GPIb-IX by interacting with the cytoplasmic domains of GPIb-IX [12]. However, the role of 14-3-3ζ in GPIb-IX-VWF interaction-induced signaling still remains controversial. We had established two CHO cell lines expressing wild type GPIb-IX (1b9) and mutant GPIb-IX replacing Ser^609^ of GPIbα with alanine (S609A) [12]. In order to investigate the role of 14-3-3ζ in GPIb-IX-VWF interaction-induced signaling, firstly, the VWF binding functions of 1b9 and S609A were assessed by flow cytometry. Consistent with the previous report [12], a certain level of VWF binding to S609A or 1b9 was detected in the present study, and there was no statistical difference in the VWF binding function between 1b9 and S609A cells (Figure S1). Then, the associations of 14-3-3ζ with GPIbα were examined in 1b9 and S609A cells by coimmunoprecipitation analysis before and after VWF binding. GPIb-IX-expressing CHO cells were firstly stimulated by ristocetin, which can induce the association of VWF with GPIbα in the presence or absence of VWF, and then were solubilized in cell lysis buffer. The lysates were immunoprecipitated with SZ2 and protein G-conjugated sepharose 4B beads, and then analyzed by SDS-PAGE and Western blot with an antibody SZ2 that specifically recognizes GPIbα and anti-14-3-3ζ antibody, respectively. As demonstrated in Figure 1, the association of 14-3-3ζ with GPIbα was obviously reduced in S609A cells both before and after VWF binding.

### 2.2. The S609A Mutation Inhibits the VWF-GPIb-IX Interaction-Induced Activation of Src Family Kinases

**Figure 2:**
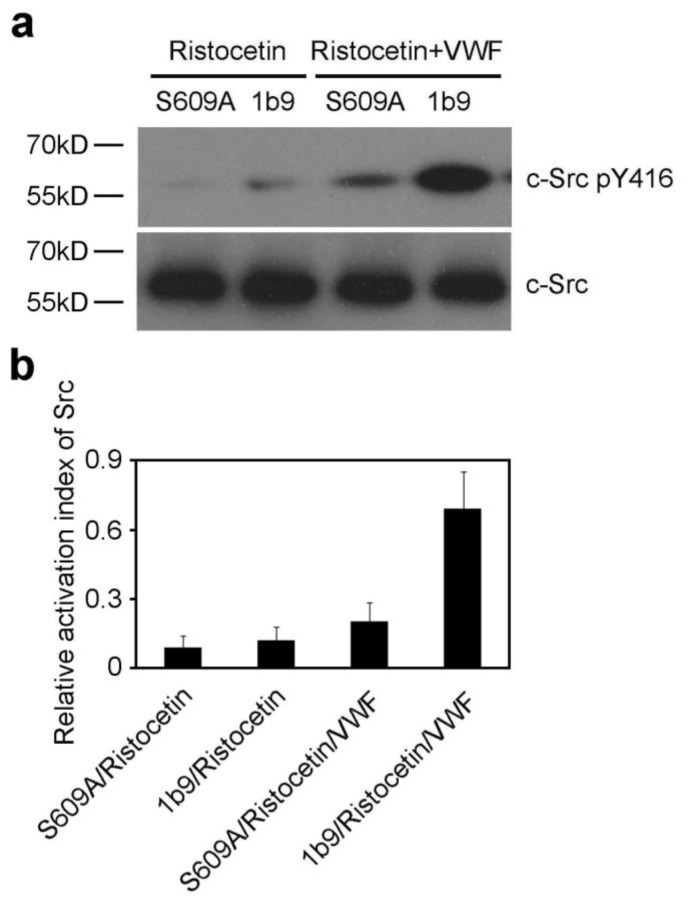
The S609A mutation inhibits activation of Src family kinases. (**a**) 1b9 or S609A cells were stimulated by ristocetin in the presence or absence of VWF, then solubilized and subjected to Western blot analysis with anti-Src and anti-phospho-Src family (pTyr416) antibody, respectively. The immunoblot is representative of 3 different experiments; (**b**) Quantitative data from 3 different experiments (mean ± SD) are demonstrated. The relative activation index of Src equals arbitrary quantitation of phospho-Src (pTyr416)/arbitrary quantitation of total Src.

The interaction of GPIbα with VWF triggers activation of mutiple protein kinases [2], such as one or more Src family kinases [18,19]. In addition, phosphorylation of tyrosine 416 (pTyr416) in the activation loop of Src upregulates its enzymatic activity. Thus, to investigate the role of 14-3-3ζ in GPIbα-VWF interaction-induced signaling, the activation of Src family kinases was analyzed. 1b9 and S609A were stimulated by ristocetin in the presence or absence of VWF, and then were lysed and subjected to SDS-PAGE and Western blot analysis with anti-Src and anti-phospho-Src family (pTyr416), respectively. The results showed that the S609A mutation resulted in inhibited activation of Src family kinases (Figure 2).

### 2.3. The S609A Mutation Inhibits the VWF-GPIb-IX Interaction-Induced Activation of PKC

**Figure 3:**
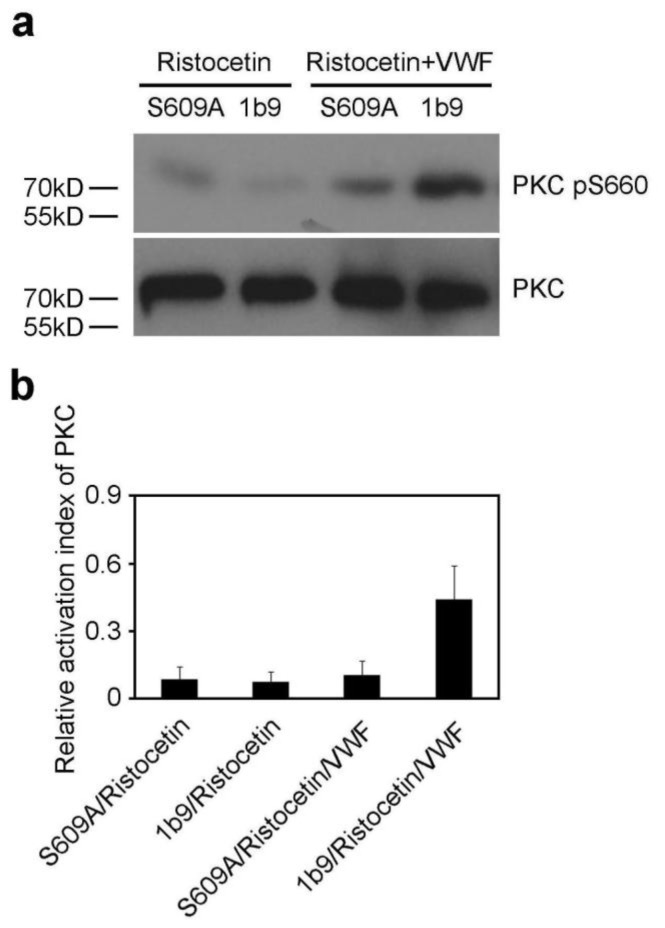
The S609A mutation blocks PKC activation. (**a**) 1b9 or S609A cells were stimulated by ristocetin in the presence or absence of VWF, and then were subjected to Western blot analysis with anti-PKC and anti-phospho-PKC (pSer660) antibody. Actin levels demonstrate similar loading. The immunoblot is representative of 3 different experiments; (**b**) Quantitative data from 3 independent experiments (mean ± SD) are shown. The relative activation index of PKC equals arbitrary quantitation of phospho-PKC (pSer660)/arbitrary quantitation of total PKC.

The interaction of GPIbα with VWF induces activation of PKC [2], which is represented as serine 660-phosphorylated PKC (pSer660). To investigate whether 14-3-3ζ is involved in activation of PKC elicited by the interaction of GPIb-IX with VWF, S609A and 1b9 cells were stimulated with ristocetin in the presence or absence of VWF, and then were subjected to PKC activation analysis. As shown in Figure 3, the S609A mutation significantly inhibited activation of PKC, indicating that the association of 14-3-3ζ with GPIbα is indispensable for GPIb-IX-VWF interaction-induced PKC activation.

### 2.4. Disruption of 14-3-3ζ Association with GPIbα Impairs Elevation of Intracellular Ca^2+^ Levels

**Figure 4:**
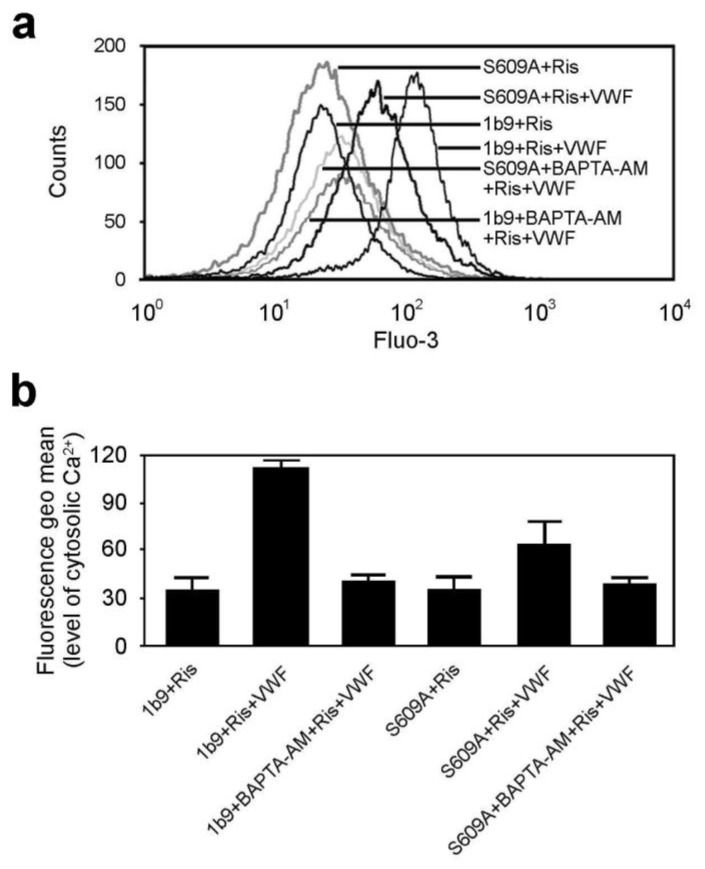
The S609A mutation inhibits elevation of intracellular Ca^2+^. (**a**, **b**) 1b9 or S609A cells were incubated with 8 μM Fluo-3/AM for 30 min at 37 °C in the dark. The external Ca^2+^ was adjusted to 1 mM, and then cells were stimulated by ristocetin in the presence or absence of VWF for 10 min at RT, and analyzed by flow cytometry. The Fluo-3/AM-loaded cells were also pre-treated with BAPTA-AM at 37 °C for 20 min before ristocetin/VWF treatment, and then intracellular Ca^2+^ levels were measured by flow cytometry. Representative histograms of Fluo3-fluorescence of cells are shown (**a**). The geometric mean fluorescence intensity of Fluo-3/AM binding is demonstrated (mean ± SD) (*n* = 3) (**b**).

The interaction of GPIb-IX with VWF triggers elevation of cytoplasmic Ca^2+^ concentrations [5,20]. Thus, the roles of 14-3-3ζ in GPIb-IX-dependent elevation of the intracellular Ca^2+^ level were investigated. As shown in Figure 4, the intracellular Ca^2+^ was significantly reduced by a membrane-permeable Ca^2+^ chelator BAPTA-AM in ristocetin-induced GPIb-IX-expressing cells, indicating that the rise in intracellular Ca^2+^ is not an artifact. Compared with wild type GPIb-IX, the S609A mutation dramatically reduced the elevation of the cytoplasmic Ca^2+^ levels in CHO cells.

## 3. Discussion

The data indicate that the S609A mutation (S609A) reduced GPIb-IX-VWF interaction-induced signaling cascades.

To investigate the role of 14-3-3ζ in GPIb-IX-VWF interaction-induced signaling, the VWF binding functions of 1b9 and S609A were firstly assessed by flow cytometry. Consistent with the previous report [12], the VWF binding function of S609A was similar to that of 1b9. Furthermore, the S609A mutation replacing Ser^609^ of GPIbα with alanine (S609A) significantly prevented the association of 14-3-3ζ with GPIbα before and after the VWF binding to GPIbα. Thus, S609A cells were employed to specify the role of 14-3-3ζ in GPIb-IX-VWF interaction-induced signaling. The data showed that GPIb-IX-VWF interaction-induced signaling cascades including activation of Src family kinase and PKC, and elevation of cytoplasmic Ca^2+^ levels were obviously reduced in the presence of the S609A mutation. Furthermore, disruption of 14-3-3ζ interaction with GPIbα by the S609A mutation induced inhibition of GPIb-IX-VWF interaction-induced phosphatidylserine (PS) exposure [21]. Since the S609A mutation did not affect the VWF binding function of GPIbα (Figure S1), the signaling inhibition by S609A was not a result of the failure of VWF binding Thus, these data indicate that in addition to the role of 14-3-3ζ in the VWF binding function of GPIb-IX, 14-3-3ζ also plays an important role in GPIb-IX-VWF interaction-induced signaling.

Both 14-3-3ζ and the regulatory p85 subunit of PI3-kinase interact with contiguous GPIbα sequences 580-590/591-610 and are associated with ristocetin/VWF interaction-induced GPIb-IX signaling [11,22]. However, pull-down experiments indicate that PI3-kinase binds to the cytoplasmic domain of GPIbα independently of 14-3-3ζ. Moreover, a 14-3-3ζ inhibitor peptide R18 showed no effect on association of GPIb-IX with GST-p85 in pull-down experiments, and GST-p85 pull-downs are not disrupted by excess 14-3-3ζ [11]. These data suggest that PI3-kinase and 14-3-3ζ interact with the *C*-terminus of GPIbα and regulate GPIb-IX-dependent signaling independently. Thus, it is reasonable to speculate that the S609A mutation may affect GPIb-IX-VWF interaction-induced GPIb-IX signaling involving 14-3-3ζ but not PI3-kinase.

There have been apparently controversial data regarding the role of 14-3-3ζ in GPIb-IX-mediated integrin activation and cell spreading [15–17]. It was reported that GPIb-IX-mediated α_IIb_β_3_ activation was inhibited in Δ591/2b3a cells co-expressing integrin α_IIb_β_3_ and mutated GPIb-IX with GPIbα truncated at residue 591 [15]. However, the data from another group showed that the interaction of 14-3-3ζ with GPIb-IX was not essential for cell spreading on VWF-coated slides and signaling transduction leading to integrin activation in GPIb-IX-expressing CHO cells [17]. Furthermore, the same group reported later that deletion of the 14-3-3ζ binding site in the *C*-terminal cytoplasmic domain of GPIbα enhanced cell spreading on VWF matrix in Δ591 cells under similar experimental conditions [16]. It was explained that the role of 14-3-3ζ in cell spreading on VWF matrix and activation of Cdc42 and Rac was secluded by the association of GPIb-IX with 14-3-3ζ. While the role of 14-3-3ζ in VWF-mediated platelet signaling and the reason for conflicting data still need to be further investigated, the data presented here indicate that 14-3-3ζ plays a key role in GPIb-IX-VWF interaction-induced signaling.

## 4. Materials and Methods

### 4.1. Antibodies and Reagents

Monoclonal antibodies SZ29 against VWF [23] and SZ2 against GPIbα [24] were described previously. Purified human VWF and botrocetin were generous gifts from Xiaoping Du (University of Illinois, Chicago, IL, USA). Ristocetin and aprotinin were purchased from Sigma (St. Louis, MO, USA). Non-essential amino acids, penicillin and streptomycin, l-glutamine, l-trans-Epoxysuccinyl-leucylamido (4-guanidino) butane (E64) were purchased from Roche Molecular Biochemicals (Indianapolis, IN, USA). Fluo-3/AM was purchased from Invitrogen Molecular Probes (Eugene, OR, USA). 1,2-*bis*(o-aminophenoxy) ethane-*N*,*N*,*N*′,*N*′-tetraacetic acid (BAPTA-AM) was purchased from Dojindo Molecular Technologies (Rockville, MD, USA). Goat anti-mouse immunoglobulin (IgG) conjugated with horseradish peroxidase (GAM-HRP), goat anti-rabbit immunoglobulin (IgG) conjugated with horseradish peroxidase (GAR-HRP), and FITC (fluorescein isothiocyanate)-conjugated goat anti-mouse IgG (FITC-GAM) were purchased from Biosource (Camarillo, CA, USA). Anti-phospho-Src family (pTyr416) rabbit polyclonal antibody was from Cell Signaling Technology (Beverly, MA, USA). Anti-Src mouse monoclonal antibody was from Upstate Biotechnology (Lake Placid, NY, USA). Anti-PKC mouse monoclonal antibody sc-17804 was from Santa Cruz Biotechnology (Santa Cruz, CA, USA). Anti-phospho-PKC (pSer660) rabbit polyclonal antibody was from BioVision (Mountain View, CA, USA (CATALOG#: 3451-100)).

### 4.2. Cell Lines Expressing Recombinant GPIb-IX and Mutants

CHO cells expressing recombinant wild-type GPIb-IX (1b9), GPIb-IX mutants (S609A) with a serine-to-alanine point mutation at Ser^609^ in GPIbα have been described previously [12].

### 4.3. Flow Cytometric Analysis of VWF Binding to GPIb-IX-Expressing Cells

1b9 or S609A cells (2 × 10^6^/mL) were stimulated by ristocetin (1.25 mg/mL) in the presence or absence of VWF (35 μg/mL) for 30 min at room temperature (RT). Pre-treated GPIb-IX-expressing cells were subjected to VWF binding analysis as described previously [12–14].

### 4.4. Coimmunoprecipitation and Western Blotting

For GPIb-IX and 14-3-3ζ association assay, GPIb-IX-expressing CHO cells including 1b9 and S609A (2 × 10^6^/mL) were firstly stimulated by ristocetin (1.25 mg/mL) in the presence or absence of VWF (35 μg/mL) for 30 min at RT, and then were solubilized in an equal volume of 2× cell lysis buffer (2% Triton X-100, 0.1 M Tris, 0.01 M EGTA, and 0.15 M NaCl, 1 mM dithiothreitol, pH 7.4) containing 0.1 mM E64 and 1 mM phenylmethylsulfonyl fluoride (PMSF). The lysates were immunoprecipitated with SZ2 and protein G-conjugated sepharose 4B beads, and then separated by sodium dodecyl sulfate-polyacrylamide gel electrophoresis (SDS-PAGE) under reducing conditions and immunoblotted with SZ2 and anti-14-3-3ζ antibody, respectively [12,14].

For Src and PKC analysis, 1b9 or S609A cells (2 × 10^6^/mL) were stimulated by ristocetin (1.25 mg/mL) in the presence or absence of VWF (35 μg/mL) for 30 min at RT. Pre-treated GPIb-IX-expressing cells were solubilized in the same cell lysis buffer and subjected to Western blot analysis under reducing conditions with anti-Src, anti-phospho-Src family (pTyr416), anti-PKC and anti-phospho-PKC (pSer660), respectively.

### 4.5. Measurement of Intracellular Ca^2+^ Levels

Intracellular Ca^2+^ concentrations were detected with the Ca^2+^-sensitive fluorochrome Fluo-3/acetoxymethyl ester (Fluo-3/AM) by flow cytometric analysis [25]. Briefly, GPIb-IX-expressing cells were incubated with 8 μM Fluo-3/AM for 30 min at 37 °C in the dark. After washing once, cells were resuspended at a concentration of 2 × 10^6^/mL. The external Ca^2+^ was adjusted to 1 mM, and then GPIb-IX-expressing cells were stimulated by ristocetin (1.25 mg/mL) in the presence or absence of VWF (35 μg/mL) for 30 min at RT, and analyzed by flow cytometry. In some experiments, Fluo-3/AM-loaded GPIb-IX-expressing cells were pre-treated with BAPTA-AM (10 μM) at 37 °C for 20 min before ristocetin/VWF treatment, and then intracellular Ca^2+^ levels were measured by flow cytometry.

## 5. Conclusions

In conclusion, the data show that disruption of 14-3-3ζ association with GPIbα by a S609A mutation reduced GPIb-IX-VWF interaction-induced signaling events, indicating that 14-3-3ζ plays an important role in GPIb-IX-VWF interaction induced signaling.

## Supplementary Material
