# Supplementary material for: Role of 14-3-3ζ in Platelet Glycoprotein Ibα-von Willebrand Factor Interaction-Induced Signaling
Source: Int J Mol Sci. 2012 May 2;13(5):5364–74. doi: 10.3390/ijms13055364 (PMC3382782; doi:10.3390/ijms13055364)
Supplement: Supplementary file 1 [file ijms-13-05364-s001.pdf]

Supplementary Material

Figure S1. S609A mutation does not affect the VWF binding function of GPIb-IX. (**a**) 1b9 or S609A cells were incubated with ristocetin in the presence or absence of VWF, and then were analyzed the VWF binding by flow cytometry. (**b**) The cells were also incubated with SZ2 to detect surface-expressed GPIb-IX levels. (**c**) Quantitative data from 3 different experiments are shown (mean ± SD). VWF binding index equals total fluorescence/background fluorescence-1.

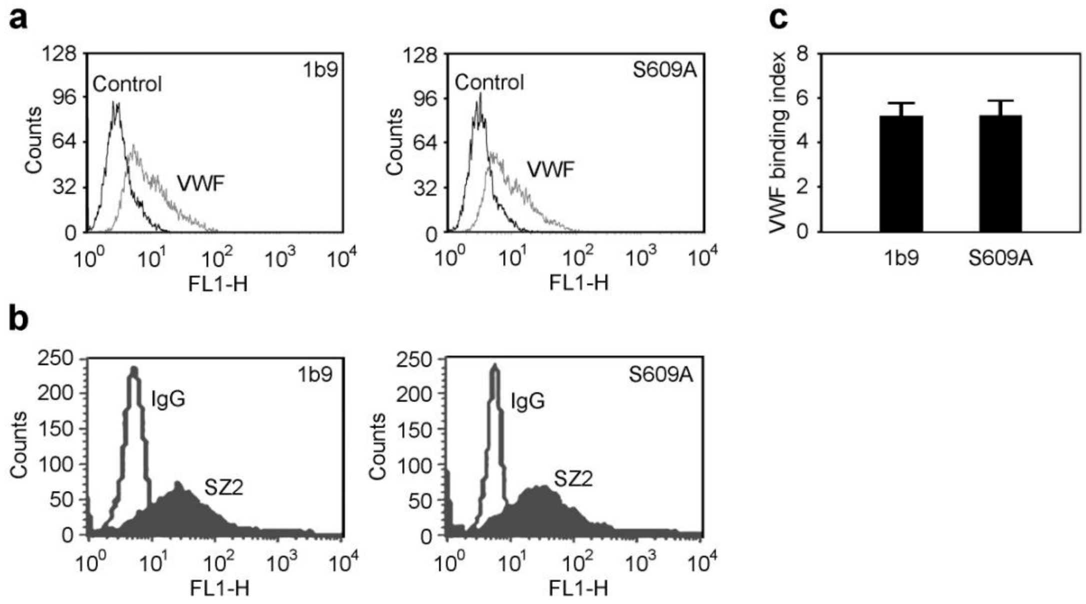

© 2012 by the authors; licensee MDPI, Basel, Switzerland. This article is an open access article distributed under the terms and conditions of the Creative Commons Attribution license (http://creativecommons.org/licenses/by/3.0/).
